# Knowledge attitudes and practices of healthcare workers on respirator fit testing and PAPR use at a university medical center

**DOI:** 10.1038/s41598-025-12507-4

**Published:** 2025-07-28

**Authors:** Fatimah Alshahrani, Abba Elgujja, Lulwa Alabdan, Jaser S. Alharbi, Ali A. Rabaan, Ibrahim Alzayid, Mohammed Alasiri, Khalid Faqihi, Samih Alanazi

**Affiliations:** 1 College of Medicine, King Saud University, Riyadh, 11362 Saudi Arabia; 2 Division of Infectious Diseases, Department of Internal Medicine, King Saud University Medical City, King Saud University, Riyadh, Saudi Arabia; 3 IPAC Department, King Saud University Medical City, Riyadh, 11362 Saudi Arabia; 4 Molecular Diagnostic Laboratory, Johns Hopkins Aramco Healthcare, Dhahran, 31311 Saudi Arabia; 5 College of Medicine, Alfaisal University, Riyadh, 11533 Saudi Arabia; 6 Department of Public Health and Nutrition, The University of Haripur, Haripur, 22610 Pakistan

**Keywords:** Respirator fit testing, Powered Air-Purifying respirator (PAPR), Healthcare professionals, Compliance, Knowledge, Attitudes, Practices, Training, Confidence, Occupational safety, Saudi Arabia, King Saud university medical City, Infection control, Respiratory protection, Demographic aspects., Health care, Preventive medicine

## Abstract

Particularly during an epidemic of infectious diseases, worker safety in healthcare depends critically on respirator fit testing and the usage of powered air-purifying respirators (PAPR). Reducing hazards requires ensuring healthcare professionals’ (HCW) knowledge, attitudes, and behaviors as well as their compliance with respiratory protection programs. There is little information on these factors in Saudi Arabian healthcare environments, which calls for targeted research. This study aimed to assess healthcare workers’ (HCW) knowledge, attitudes, and practices (KAP) regarding respirator fit testing and powered air-purifying respirator (PAPR) use at King Saud University Medical City (KSUMC) which is referred to as ‘the medical center’ throughout the paper. Specifically, it sought to identify gaps in policy understanding and training, evaluate compliance and confidence levels, and examine how demographic variables influence these outcomes. A total of 204 HCWs from different departments and hospitals around the medical center participated in cross-sectional research. Structured surveys measuring demographic variables, knowledge, attitudes, training experience, and compliance with fit testing and PAPR use gathered data. While chi-square tests and correlation analysis look at relationships between variables, descriptive statistics compile the demographic traits and survey answers. With SPSS, version 27, all the statistical tests were run with a significance threshold of α = 0.05. With respirator fit testing, the results revealed a high compliance rate—93.4%. Nurses had the best rates of compliance and confidence. However, demonstrating a large knowledge gap, only 6.9% (N-36) of the respondents knew about quantitative fit assessment techniques. Among the 82.2% (N-168) of HCWs who reported PAPR usage training, 48% (of N-168) received consistent instructions. While 14.8% (of N-168) of the respondents reported poor confidence, suggesting room for development, PAPR use was rather high—85.2% (N-204). Significant correlations were found between demographic variables and compliance, training, and confidence levels (*p* < 0.05). In particular, a negative connection between PAPR usage (*r* = -0.287, *p* = 0.01) and confidence in fit testing indicated possible specialized effects. This study highlights the need for thorough and consistent respiratory protection training courses for different HCW profiles. Respiratory protection measures at KSUMC may be strengthened even further by addressing knowledge gaps, increasing hands-on training, and strengthening policy communication to guarantee HCW safety and preparedness.

## Introduction

Healthcare workers (HCWs), who are often exposed to workplace dangers, including airborne infections, are front-line in the fight against infectious illnesses. Essential measures to guarantee the safety and health of HCWs in high-risk workplaces include respiratory fit testing and the use of powered air-purifying respirators (PAPRs)^1^. During epidemics such as SARS, H1N1, and COVID-19, where airborne transmission is a major factor, proper respiratory protection is especially vital^2^. Nevertheless, in many healthcare environments, attaining compliance and guaranteeing proper use of respiratory protection tools remain difficult^3^.

Effective protection against airborne dangers is provided by a good seal between the respirator and the user’s face, which is ensured by respirator fit testing^4^. Fit testing may be performed mostly via qualitative and quantitative approaches. Whereas the latter measures leakage surrounding the respirator via machines, the former depends on the user’s sensory identification of test agents^5^. Both approaches are extensively employed; however, knowledge gaps and uneven training often result in incorrect application and lower effectiveness^6^. Furthermore, a major factor influencing compliance is HCWs’ trust in their capacity to apply respiratory protection successfully^7^.

Often advised for HCWs who fail fit testing or when the respirator supply is restricted, PAPRs are advanced respiratory protective devices that provide increased protection^8^. Despite its advantages, PAPR adoption faces challenges, including poor training, perceived complexity, and a lack of user-friendly design^1^. These difficulties highlight the need for customized training courses to increase confidence and competency in PAPR use^9^.

In particular, at major academic medical centers such as this, Saudi Arabia has little information on the knowledge, attitudes, and practices of HCWs with respect to respiratory protection. Research on comparable environments already in publication indicates that compliance with fit testing and PAPR use is strongly influenced by profession, department, and years of experience^10^. Furthermore, differences in training and confidence among demographics might lead to unequal application of respiratory protection policies^11^. Designing successful treatments and policies thus depends on an exploration of these factors.

The objectives of this study are:^1^ to evaluate HCWs’ knowledge, attitudes, and practices concerning respirator fit testing and PAPR use;^2^ to identify training and policy knowledge gaps; and^3^ to examine how demographic characteristics (e.g., profession, department, experience) influence compliance, training, and confidence. These insights aim to support improvements in institutional respiratory protection programs.

## **Methodology**

This study employed a cross-sectional, descriptive design to assess the knowledge, attitudes, and practices (KAP) of HCWs regarding respirator fit testing and PAPR use. The design was chosen for its efficiency in capturing a snapshot of current practices and beliefs within a defined timeframe without manipulating variables. Data were collected over an eight-week period from October to December 2024. The cross-sectional approach allowed for the identification of associations between demographic factors and outcomes (e.g., compliance, confidence, training) while minimizing time, cost, and ethical constraints typically associated with longitudinal or experimental designs.

This study offers a thorough overview of the approach used in the study evaluating HCWs at the medical center for their knowledge, attitudes, and behaviors related to respirator fit testing and PAPR use. To ensure the validity and dependability of the study, the approach describes the study design, demographic and sampling strategies, data collection methodologies, instruments, processes, and data analysis approaches.

### Study design

The knowledge, attitudes, and behaviors of HCWs regarding respirator fit testing and PAPR use were gathered in this cross-sectional design study. Given the efficiency in capturing a snapshot of correlations between demographic characteristics and intended outcomes within the limitations of time and money, a cross-sectional design was selected above longitudinal or experimental designs. Unlike longitudinal studies, which require repeated measurements over long periods, this method allows for the instantaneous discovery of gaps and correlations important for guiding training and policy changes. Although the experimental designs were strong in proving causation, ethical issues and the observational character of the study focus judged them unworkable.

### Study setting

The study was conducted at the medical center, a major academic and tertiary care healthcare institution located in Riyadh, Saudi Arabia. the medical center serves as the teaching and training hub for King Saud University’s College of Medicine and comprises several hospitals and facilities, including King Khalid University Hospital (KKUH), King Abdulaziz University Hospital (KAUH), the Dental University Hospital (DUH), and the General University Clinic (GUC). These institutions offer a wide range of inpatient, outpatient, surgical, and diagnostic services. As a multidisciplinary and high-volume medical center, KSUMC provides an ideal setting for investigating healthcare workers’ knowledge and practices related to respiratory protection, especially in the context of infection control and occupational safety.

### Study population and sampling

The study population consisted of HCWs employed at the medical center, including nurses, physicians, allied health professionals, technicians, engineers, and support staff. These professionals were selected based on their potential exposure to airborne hazards and/or their use of respirators or powered PAPRs during clinical or support activities.

#### Inclusion Criteria

- Current employment at one of the medical center’s affiliated hospitals or clinics during the study period (October–December 2024).
- Clinical or operational roles requiring the use of respirators or PAPRs.
- Willingness to participate and provide informed consent.

#### Exclusion Criteria

- Administrative staff or employees not involved in patient care or infection-prone environments.
- HCWs on extended leave or unavailable during the data collection period.

A non-probabilistic purposive sampling strategy was employed to ensure the inclusion of participants from various departments, professions, and levels of exposure to respiratory protection protocols. This approach was chosen to capture diverse perspectives and to ensure representation of HCWs most relevant to the study’s objectives.

The minimum required sample size was calculated using Raosoft’s online sample size calculator (http://www.raosoft.com/samplesize.html), assuming a 95% confidence level, a 5% margin of error, and a response distribution of 50%, which is a conservative estimate when prevalence is unknown. Based on an estimated total HCW population of approximately 500 across the medical center’s major facilities, the required sample size was 218. A total of 204 complete responses were ultimately obtained and analyzed, representing a high response rate (93.6%) for a voluntary cross-sectional survey.

### Data collection instrument

Data were collected using a structured, self-administered questionnaire specifically developed for this study, based on a comprehensive review of the literature and adapted in part from validated instruments used in prior studies on respirator fit testing and PAPR usage^1,4,7^. The instrument was designed to assess healthcare workers’ knowledge, attitudes, and practices (KAP) in alignment with infection control and occupational safety principles.

The questionnaire consisted of five main sections:

1. Demographic Information (e.g., age, gender, occupation, department, years of experience, and hospital site);
2. Knowledge about respirator fit testing, PAPR components, usage protocols, and policy understanding;
3. Attitudes toward the importance, usability, and perceived confidence in fit testing and PAPR use;
4. Practices, including frequency of fit testing, training received, compliance behavior, and routine use;
5. Training Needs and Preferences, using open- and closed-ended items.

The initial draft was reviewed by a panel of five subject-matter experts, including infection prevention and control (IPAC) specialists, an occupational health physician, and two epidemiologists from Medical City. These experts assessed the questionnaire for content validity, relevance, and clarity. Modifications were made based on their feedback to enhance readability, domain coverage, and item alignment with current practices and policies.

Pilot testing was conducted with 20 healthcare workers from various clinical departments at the medical center to assess clarity, timing, and item interpretation. Feedback from the pilot led to minor wording adjustments, particularly in items related to Likert-scale anchors and institutional policy descriptions.

Reliability was assessed using Cronbach’s alpha across the key domains:

- Knowledge domain: α = 0.78.
- Attitude domain: α = 0.81.
- Practice domain: α = 0.85.
- These results indicate good internal consistency for the instrument.

The final questionnaire was made available in both digital and paper formats, and participants were given two weeks to complete it, with reminders sent periodically to enhance response rates.

### Data collection procedure

Data collection was conducted over an eight-week period from October to December 2024. The finalized questionnaire was distributed to eligible healthcare workers through a mixed-mode approach, including both digital (email links via Google Forms) and paper-based formats to maximize accessibility across departments and shifts.

Targeted announcements were sent via internal institutional email and posted in staff lounges and nursing stations. Departmental heads were contacted to encourage participation without coercion. Participants were informed about the purpose of the study, estimated time to complete the survey (10–15 min), and their right to withdraw at any point.

To ensure broad representation and adequate response volume, participants were given two weeks to complete the questionnaire, with weekly reminders issued to non-responders through email and posted bulletins. Staff who opted for paper-based surveys submitted their responses via sealed drop boxes placed in designated administrative offices. These responses were collected by trained study personnel, digitized manually, and verified through double data entry.

A total of 204 valid and complete responses were received, from an estimated 218 targeted participants, yielding a 93.6% response rate. Minor missing values were identified in some demographic fields (< 2%) and were addressed using multiple imputation techniques to avoid list-wise deletion and preserve the dataset’s integrity.

Anonymous participation was ensured by not collecting any personally identifiable information. Digital responses were submitted through secure, password-protected forms with no IP tracking enabled. Paper surveys were coded without names or identifiers. All data were stored on encrypted, access-restricted institutional servers, accessible only to the principal investigator and the data analysis team.

### Context of research

One of Riyadh, Saudi Arabia’s largest academic healthcare institutions, the medical center, performed the study. Among the various hospitals included in the medical center are Dental University Hospital (DUH), General University Clinic (GUC), King Khalid University Hospital (KKUH), and King Abdulaziz University Hospital (KAUH). These institutions teach hospitals for medical students and trainees and offer various clinical settings. The inclusion of many sites guaranteed a thorough representation of HCWs working for the university.

### Study subjects

This study focused on HCWs with several professional capacities, such as nurses, doctors, lab technicians, engineers, physiotherapists, and support personnel. The eligibility requirements were as follows: work at the medical center during the research period. Participation in clinical or support roles requiring respirators or PAPRs. Openness to provide informed permission.

The exclusion criteria were individuals not active in roles requiring respiratory protection and HCWs on extended leave throughout the research period. These standards guaranteed that the sample reflected the active workforce at the medical center actively involved in patient care and support programs. The inclusion of HCWs from several departments and professional roles allowed the study to gather a wide spectrum of experiences and practices, hence improving the generalizability of the results to other like-minded healthcare environments.

### Methods of sampling

Participants from every the medical center facility were sought via a purposeful sampling technique. This strategy was selected to specifically target HCWs with different degrees of respiratory protection measure exposure, therefore guaranteeing a variety of experiences and behaviors. By concentrating on particular subgroups, notably those employed in departments highly exposed to airborne hazards, the study reduced the possibility of under-representation of important viewpoints. Moreover, by prioritizing inclusion among many professions and departments, intentional sampling helps reduce possible prejudices. To reach a 95% confidence level with a 5% margin of error, the target sample size was calculated on the basis of the population of healthcare staff at King Saud University Medical City. A total of 204 HCWs, spanning a wide spectrum of departments and professional specialties, participated in the study.

### Data collection

Data were gathered via a structured, self-administered questionnaire created on the basis of current research and validated instruments applied in such investigations. The questionnaire has five sections: Demographics, inquiries concerning participants’ age, gender, occupation, department, and years of experience. Knowledge: Questions on institutional policies, PAPR components, and respirator fit testing techniques. Likert-scale questions measuring participants’ opinions of the user-friendliness and relevance of respiratory protection. Practices: Enquiring adherence to policies and frequency of training as well as fit testing and PAPR use. Multiple-choice and open-ended questions highlight areas lacking in preferred approaches to instruction and training requirements.

The content validity of the questionnaire was guaranteed by a panel of occupational health and infection control specialists. Using Cronbach’s alpha, dependability was evaluated during the pilot test among twenty HCWs, with an overall value of 0.82, reflecting great internal consistency. Pilot comments helped guide changes meant to increase applicability and clarity.

### Data collection procedures

Data collection was conducted from October to December 2024 over eight weeks. The actions were as follows: Approval was obtained from the Institutional Review Board (IRB) at King Saud University. The research followed ethical standards, including voluntary involvement and anonymity. The participants were gathered via announcements, emails, and posters in departments. Attempts were made to guarantee inclusion by reaching HCWs in every division. The participants were advised of the research goals, methods, and freedom to stop at any point. For paper submissions, consent was gathered either digitally or on signed forms. The participants had two weeks to finish the questionnaire. Periodically delivered reminders were meant to maximize response rates. Those that chose paper surveys underwent a methodical follow-up process. To preserve consistency in data quality, survey organizers ensured that the completed paper forms were securely gathered, digitalized right away, and cross-checked for accuracy via the digital data set.

### Institutional review board statement

This study was conducted in accordance with the ethical principles outlined in the Declaration of Helsinki. Ethical approval was obtained from the Institutional Review Board of King Saud University College of Medicine, Riyadh, Saudi Arabia (IRB No. KSU-2024-199). The study was categorized as a minimal-risk observational study and was exempt from clinical trial registration, as it did not involve any intervention or collection of biological specimens. Nonetheless, all study procedures were reviewed and approved by the IRB to ensure ethical compliance and participant protection. All participants were provided with detailed information regarding the study’s purpose, procedures, and their rights before participation. Informed consent was obtained from each participant either electronically (via a digital acknowledgment checkbox in the online survey) or in writing (via signed forms accompanying the paper-based survey). Participation was entirely voluntary, with no incentives offered, and participants were free to withdraw at any time without any consequences. The survey was anonymous; no personally identifiable information was collected. Data were stored securely on encrypted, access-restricted institutional servers, accessible only to the principal investigator and authorized research personnel. These measures ensured participant confidentiality and compliance with ethical standards for human research.

The study was approved by the Institutional Review Board (IRB) of King Saud University College of Medicine, Riyadh, Saudi Arabia (IRB No. KSU-2024-199). All procedures were conducted in accordance with the Declaration of Helsinki. The study was classified as minimal risk and did not require clinical trial registration.

### Informed consent

was obtained from all participants. For online surveys, consent was provided electronically via checkbox. For paper-based surveys, signed written consent was obtained. Participation was voluntary and anonymous, and no identifiable data were collected. Data were stored securely on encrypted servers with access restricted to the research team.

### Data analysis

SPSS version 27 was used to examine the data. Survey responses and demographic traits were compiled via descriptive statistics, including frequencies, percentages, means, and standard deviations. Relationships and group differences were investigated via inferential statistical tests.

To ensure the appropriate application of statistical tests, variables were classified according to standard measurement levels. Categorical variables included department, hospital affiliation, occupation, and compliance status (e.g., fit testing performed or not, type of respirator used). Ordinal variables comprised Likert-scale responses assessing knowledge, confidence, perceived user-friendliness, and training adequacy, typically measured on 3- to 5-point scales. Continuous variables included age and years of professional experience. This classification informed the selection of statistical methods such as chi-square tests for categorical comparisons, correlation analyses for ordinal data, and t-tests or ANOVA for continuous variable group differences.

Likert-scale responses (e.g., confidence in respirator use, perceived training adequacy, agreement with institutional policies) were primarily treated as ordinal data and analyzed accordingly using non-parametric correlation (Spearman’s rho) and chi-square tests, unless otherwise specified. In some analyses, Likert-scale responses were recoded into dichotomous categories for clarity (e.g., “agree” vs. “disagree” or “confident” vs. “not confident”) when exploring group comparisons. No composite scores or standardized indices were created, as the study aimed to assess each domain (knowledge, attitudes, and practices) independently. The decision not to use composite scores was also driven by the heterogeneity of item content and the focus on domain-specific descriptive insights.

To investigate the relationships between categorical variables such as hospital affiliation, department, occupation, and fit testing compliance, chi-square tests were used. Through correlation analysis, one may evaluate links between PAPR use and respirator fit testing confidence levels. The mean scores of knowledge, attitudes, and practices among the demographic groups were compared via independent t tests and ANOVA.

Missing data were resolved via many imputation methods. This method replaces missing values with estimations generated from observed data patterns, therefore guaranteeing that every analysis is based on a complete data set. This helped to preserve the integrity of the findings and reduce any biases from inadequate answers. When a significance level of α = 0.05 was applied, p values less than 0.05 were regarded as statistically significant.

Descriptive statistics were conducted to summarize the key characteristics of the study population and responses. Frequencies and percentages were used to describe categorical variables such as hospital affiliation, department, professional role, training status, and compliance with fit testing or PAPR usage. Ordinal variables—such as Likert-scale responses related to confidence, user-friendliness, perceived importance, and policy awareness—were also summarized using frequencies and percentages. Continuous variables such as age and years of professional experience were summarized using means and standard deviations. These descriptive statistics provided the foundational understanding of demographic distributions and informed the selection of appropriate inferential tests.

Data analysis was conducted using IBM SPSS Statistics version 27. Descriptive statistics (frequencies, percentages, means, and standard deviations) summarized demographic variables and questionnaire responses. Inferential analyses were performed based on data type and distribution: chi-square tests were used for categorical comparisons (e.g., hospital affiliation and fit testing status), t-tests and ANOVA were used for comparing continuous outcomes across groups (e.g., age or years of experience), and Spearman’s rho was employed for ordinal variables (e.g., Likert-scale ratings of confidence and usability). Normality assumptions were checked using Shapiro-Wilk tests and visual inspection of histograms and Q-Q plots before applying t-tests or ANOVA. For missing data (less than 2% in some demographic variables), we applied Multiple Imputation by Chained Equations (MICE) with five imputations to preserve statistical power and minimize bias. The imputation model included all key demographic and outcome variables (e.g., age, gender, department, training status, compliance, and confidence scores). Statistical significance was set at α = 0.05 for all tests.

Statistical analyses were performed using IBM SPSS Statistics version 27. Variables were classified by level of measurement: categorical (e.g., profession, hospital affiliation), ordinal (e.g., Likert-scale items on confidence and usability), and continuous (e.g., age, years of experience). Descriptive statistics summarized the sample. Inferential tests included chi-square tests for categorical comparisons, t-tests and ANOVA for continuous group differences, and Spearman’s rho for ordinal correlations. Normality was checked using the Shapiro-Wilk test and Q-Q plots. Missing data (< 2%) were handled using Multiple Imputation by Chained Equations (MICE), with five imputations including key demographic and outcome variables in the imputation model.

### Guarantee validity and reliability

These steps were taken to guarantee the quality and dependability of the research results. Experts examined and improved the questionnaire and then tested it through pilot research to establish dependability via Cronbach’s alpha. The use of strong statistical techniques and imputation for missing data guarantees correct and dependable outcomes. Triangulation: To improve robustness and contextualize the results, the findings were cross-checked with previously published material. The surveys were taken anonymously to reduce any answer biases, and the questionnaire was developed with neutral language to prevent suggestive phrasing or leading questions. These steps guaranteed that the answers revealed the participants’ actual knowledge, attitudes, and behavior.

### Ethical issues

The study followed the ethical standards specified in the Declaration of Helsinki. By providing survey answers with distinct identities, participants’ anonymity and confidentiality were preserved. The participants’ rights—including their right to stop the research at any moment without penalty—were well recognized by them, and efforts were taken to guarantee them. Written permission forms and spoken briefings at recruiting events provide thorough descriptions of the research goals, methods, and ethical issues. Consent was confirmed via digital acknowledgment for online replies or signed forms for paper entries. Only the study team could access data that were kept safe on encrypted servers. There were no hazards for the participants; thus, no incentives were given to prevent coercion and guarantee ethical behavior by means of avoiding danger.

### Limitations of the methodology

Although the research approach and design were strong, certain constraints were admitted: Dependency on self-reported answers could cause social desirability bias and memory. For more objective and triangulated data collection, future research might use mixed-method techniques or observational methodologies.

Purposive sampling might restrict generalize-ability to populations or different healthcare environments. Using stratified or random sample methods in future studies will help improve the relevance of the results. Addressing these constraints in further studies would help improve the knowledge of respiratory protection strategies among healthcare professionals. Combining qualitative interviews with polls, for example, could offer a more thorough understanding of compliance obstacles and incentives. The comparative relevance and generalize-ability of the results might also be improved by performing such research in several healthcare systems. One may argue for the more general relevance of random sampling techniques.

Notwithstanding these constraints, the approach provides a thorough framework to investigate knowledge, attitudes, and practices among HCWs at the medical center, generating insightful information for improving respiratory protection policies. This work provides a strong basis for suggestions meant to improve occupational safety and training programs inside the healthcare sector by combining strict statistical analysis with morally sound and context-specific data collection techniques.

The methodological design and analysis framework outlined above ensured a robust approach to evaluating knowledge, attitudes, and practices related to respiratory protection. The following section presents the study’s findings.

## Results

The sample comprises 204 HCWs of the medical center. After King Abdulaziz University Hospital (KAUH) at 22.1% (N-45), most of the responders 60.8% (N-124) work at King Khalid University Hospital (KKUH). Smaller percentages work at the general university clinic (GUC) (2.0%) and dental university hospital (DUH) (11.3% (N-23)).

With 65.7% (N-134) of the sample, nursing department staff constitute the largest group across departments. Additional important departments include “Others” (13.2%(N-27)), Medicine (8.3%(N-17)), and Surgery (6.9%(N-14)). With 0.5% of the sample each, the remaining departments—engineering, laboratory, rehabilitation, and support services—each account for.

Professionally, nurses predominated in 84.3% (N-172)of the sample. While 5.9%(N-12) belong to the “Others” category, other occupations include doctors (4.9%(N-10)). Each representing 0.5% (N-1 each) of the sample is composed of physiotherapists and engineering workers.

The staff members’ experience ranges widely. Working at KSUMC for more than one year but less than five years makes up the largest group (34.8%(N-71)). Those with 5–10 years of experience (19.6%(N-40)) and those with 10–15 years (19.1%(N-39)) come next. Among the sample, 14.7%(N-30) were staff members with more than 15 years of experience; 7.8%(N-16) were staff members with less than one year of experience.

With a good range of experience levels, this sample mostly consists of nurses from KKUH, thereby offering a strong platform for assessing the knowledge and attitudes toward respirator fit testing and PAPR use among healthcare professionals at KSUMC. Table 1.

**Table 1:** Analysis of staff distribution and professional experience across healthcare settings at KSUMC.

Staff information	Frequency(*N*)	Percentage (%)
Hospital		
DUH	23	11.3%
GUC	4	2.0%
KAUH	45	22.1%
KKUH	124	60.8%
Department		
Engineering	1	0.5%
Laboratory	1	0.5%
Medicine	17	8.3%
Nursing	134	65.7%
Others	27	13.2%
Rehabilitation	1	0.5%
Support service	1	0.5%
Surgery	14	6.9%
What is your profession at KSUMC?		
Engineering Staff	1	0.5%
Nurse	172	84.3%
Others	12	5.9%
Physician	10	4.9%
Physiotherapist	1	0.5%
How long have you been working in a healthcare setting at KSUMC? (Choose one answer)		
Less than 1 year	16	7.8%
More than 1 year but less than 5 years	71	34.8%
More than 10 years but less than 15 years	39	19.1%
More than 15 years	30	14.7%
More than 5 year but less than 10 years	40	19.6%

Table 2 presents a summary of the descriptive statistics for the study population, including demographic variables (age, gender, occupation, department, years of experience) and key outcome indicators related to knowledge, attitudes, and practices (KAP) toward respirator fit testing and PAPR use. Categorical variables are expressed as frequencies and percentages, while continuous variables are reported using means and standard deviations. This overview provides essential context for the interpretation of subsequent inferential analyses.

**Table 2:** Descriptive statistics of study participants (*N* = 204).

Variable	Category/measure	Frequency (*n*)	Percentage (%) / Mean ± SD
Age (years)	–	–	34.6 ± 8.2
Gender	Male	76	37.3%
	Female	128	62.7%
Occupation	Nurse	172	84.3%
	Physician	10	4.9%
	Others	22	10.8%
Department	Nursing	134	65.7%
	Medicine	17	8.3%
	Surgery	14	6.9%
	Others	39	19.1%
Years of experience	< 1 year	16	7.8%
	1–5 years	71	34.8%
	5–10 years	40	19.6%
	10–15 years	39	19.1%
	> 15 years	30	14.7%
Fit testing compliance	Yes (Valid Certificate)	183	93.4%
	No or Expired/Unfit	13	6.6%
PAPR usage training	Received Regular Training	94	48.0%
	Irregular or No Training	102	52.0%
Knowledge (Fit Testing Purpose)	Correctly Identified	176	86.3%
Confidence in PAPR Use	High (4 or 5 on Likert Scale)	98	50.0%
Perception of PAPR Usability	Highly User Friendly	149	76.0%

With almost perfect conformity with fit testing criteria, the great majority of respondents—93.4%(N-183)—have been fit tested and carry a valid certificate. With 61.8%(N-110), the qualitative approach—using a hood—was employed more often than the quantitative method was—38.2%(N-68). Most respondents—93.6%(N-178)—passed their prior fit test; 68.9%(N-131) passed the qualitative test, and 24.7%(N-47) passed the quantitative test.

With respect to respirator models, 3 M 8210 was the most often used (39.8%(N-78)), followed by Halyard types. While 24.5% of the respondents received training but not consistently, a sizable portion—59.7%(N-117)—said that they routinely received appropriate testing training. Most (86.2%(N-170)) said they had great confidence—that is, 4 or 5 on a 5-point scale—in their awareness of the need for consistent fit testing.

Appropriate practices are well understood; 68.4%(N-14) of respondents correctly noted that seal inspection should be performed prior to every usage. With respect to respirator usage, nevertheless, the KSUMC policy is not entirely clear; only 48.4% (N-93) of respondents correctly identified the single-use regulation.

Strong knowledge of the fit testing objective is evident; 86.3% (N-176)of the respondents correctly identified it guarantees a good seal between the respirator and the user’s face. Although less familiar with the quantitative method (6.9%(N-14)), most respondents—69.1%(N-141)—are aware of the qualitative fit test approach.

Most (88.3%(N-173)) understood that fit testing should be performed every two years, and 91.3%(N-179) understood that severe facial changes call for retesting. Nearly all the respondents (95.9%(N-188)) agreed that consistent fit testing is extremely necessary to guarantee appropriate respiratory protection.

The results indicate a high degree of awareness, compliance, and favorable opinions of fit testing among HCWs at KSUMC overall. Nevertheless, there are several areas where knowledge can be lacking, especially with respect to the unique laws on respirator usage and the many techniques of fit testing. With 86.2%(N-169) of the respondents saying that the IPAC software for respiratory fit testing at KSUMC is extremely user friendly, their opinions are generally good.

These results provide a good basis for respiratory protection policies at KSUMC as well as areas where focused education and training may improve awareness and compliance even further. See Table 3.

**Table 3:** Assessment of knowledge and attitudes about regular fit testing for respirators.

Assessment of knowledge and attitudes about regular fit testing for respirators	Frequency(*N*)	Percentage(%)
Have you been fit tested?		
I do not remember	2	1.0%
No, never been fit tested	7	3.6%
Yes, but my certificate has expired	4	2.0%
Yes, my certificate is still valid	183	93.4%
If you did a fit test before, which method was used for you?		
Qualitative (use of hood)	110	61.8%
Quantitative (use of machine)	68	38.2%
What was the result of your previous fit test? (Choose one answer) Answer this if your selected answer to the question above was 1 or 2.		
Failed Qualitative, but passed Quantitative	1	0.5%
Failed Quantitative, but passed qualitative	1	0.5%
Failed, Quantitative	2	1.1%
I am not sure or cannot remember	5	2.6%
I have not been fit-tested before	3	1.6%
Passed, Qualitative	131	68.9%
Passed, Quantitative	47	24.7%
If you have failed the previous test, which of the following was the reason? (check all that apply)		
I have beard	9	4.4%
I have dentures	2	1.0%
I recently lost/gained weight	4	2.0%
I recently have facial surgery	2	1.0%
I do not know/do not remember	4	2.0%
Not applicable/I passed the fit test	132	64.7%
What is your current Respirator model and size as stated on your fit test card? (Choose one answer)		
3 M 1804	5	2.6%
3 M 1805	2	1.0%
3 M 1805s	1	0.5%
3 M 1860	11	5.6%
3 M 1860s	4	2.0%
3 M 1870+	11	5.6%
3 M 8210	78	39.8%
3 M 9320+	13	6.6%
3 m 1804s	2	1.0%
BYD Care De2322	12	6.1%
Halyard 46,727	19	9.7%
Halyard 46,827	15	7.7%
I do not remember or my card is lost	7	3.6%
I was not tested or failed the test	6	3.1%
PAPR	10	5.1%
Have you received any training that include information on fit test? (Choose one answer)		
No, and I am not interested in receiving training	8	4.1%
No, but I would like to receive training	23	11.7%
Yes, but not regularly	48	24.5%
Yes, regularly	117	59.7%
How confident are you in your knowledge of the importance of regular fit testing for respirators? (Choose one answer)		
1	5	2.6%
3	21	10.7%
4	58	29.6%
5	112	57.1%
How often do you believe seal checking for respirators should be conducted? (Choose one answer)		
Before each use	134	68.4%
Once a month	12	6.1%
Once a year	30	15.3%
Only if the respirator is visibly damaged or the user experiences discomfort	20	10.2%
Which of the following responses represents the true position of KSUMC policy on the proper use of Respirator? (Choose one answer)		
I can continue to use a respirator for as long as possible provided that I can properly store it.	23	12.0%
I can extend use of one respirator throughout the shift unless it becomes soiled, damaged or contaminated	53	27.6%
I can use one respirator during the whole shift, regardless	23	12.0%
I can use one respirator only once, and then discard it after use.	93	48.4%
What is the purpose of fit testing for respirators? (Select all that apply)		
To ensure a proper seal between the respirator and the user’s face	176	86.3%
To determine the user’s comfort level while wearing the respirator	6	2.9%
To assess the respirator’s filtration efficiency	9	4.4%
To identify any issues with the respirator that may affect its performance	5	2.5%
Are you aware of the different methods used for fit testing? (Select all that apply)		
Qualitative fit test	141	69.1%
Quantitative fit test	14	6.9%
User seal check	16	7.8%
No, I am not aware of any fit testing methods	25	11.8%
How often should fit testing for respirators be regularly repeated? (Choose one answer)		
Every 2 years	173	88.3%
Every 6 months	4	2.0%
Every year	11	5.6%
Only if the user’s facial features change significantly	8	4.1%
What changes necessitate repeating the fit test before the regular validity period? (Choose one answer)		
A). Weight loss/gain	6	3.1%
B). Facial surgery	5	2.6%
C). Loss of dentures	1	0.5%
D). None of the above	5	2.6%
E). A, B & C	179	91.3%
Do you believe that regular fit testing for respirators is important in ensuring proper respiratory protection for HCWs? (Choose one answer)		
No, not truly	2	1.0%
Yes, absolutely	188	95.9%
Yes, to some extent	6	3.1%
Do you agree that the IPAC program for respiratory fit testing at KSUMC is user-friendly? (Choose one answer)		
a) Yes, absolutely	169	86.2%
b) Yes, somewhat	19	9.7%
c) Not truly	8	4.1%

This information shows the results of chi-square tests looking at the link between certain demographic variables and whether the medical center’s HCWs have been fit tested for respirators. Across all the demographic factors examined, the study revealed notable correlations.

Fit testing status (φ^2^ = 18.835, *p* = 0.027) reveals a notable link according to hospital affiliation. In particular, all 45 KAUH respondents had legitimate fit test certificates; other hospitals had more different results. This suggests that variations may existing compliance or fit testing methods among many hospital facilities within the medical center.

Fit testing status (φ^2^= 111.812, *p* < 0.001) shows a rather strong correlation with department affiliation. While certain departments, such as laboratory and support services, offer more variable replies, the nursing department has the largest number of genuine certifications. This can point to variations in fit testing accessibility or priority between many hospital divisions.

Furthermore, profession has a very significant correlation with fit testing status (φ^2^ = 124.288, *p* < 0.001). While other occupations exhibit more varied replies, nurses almost always report having legitimate fit test certificates (165 out of 172). This implies that, among nursing professionals, fit testing might be carried out or enforced more often than in other healthcare professions.

Fit testing status (φ^2^ = 33.542, *p* = 0.001) is strongly correlated with length of work experience at KSUMC. While those with less than one year of experience exhibit more varying replies, HCWs with 1–5 years of experience constitute the highest proportion of valid certifications. This would suggest that, albeit throughout the first few years of work, fit testing is more likely to take place following the first on-boarding process.

These findings show overall notable differences in fit testing status among several demographic variables within the KSUMC. Strong correlations imply that whether an HCW has undergone fit testing depends greatly on their department, profession, job experience, and hospital affiliation. These results can be helpful in identifying areas where standardizing across the healthcare system or improving fit testing procedures may be needed. See Table 4.

**Table 4:** Respirator fit Testing.

		Have you been fit tested				Chi square	P value
		I do not remember	No, never been fit tested	Yes, but my certificate has expired	Yes, my certificate is still valid		
Hospital	DUH	1	2	0	20	18.835^a^	0.027
	GUC	0	0	1	3		
	KAUH	0	0	0	45		
	KKUH	1	5	3	115		
Department	Laboratory	1	0	0	0	111.812^a^	0.000
	Medicine	0	0	0	1		
	Nursing	0	1	1	15		
	Others	0	3	1	130		
	Support service	0	2	1	24		
	Surgery	0	0	0	1		
What is your profession at KSUMC	Nurse	1	3	3	165	124.288^a^	0.000
	Others	0	1	0	11		
	Physician	0	3	1	6		
How long have you been working in a healthcare setting at KSUMC?	Less than 1 year	0	4	0	12	33.542^a^	0.001
	More than 1 year but less than 5 years	2	0	0	69		
	More than 10 years but less than 15 years	0	0	1	38		
	More than 15 years	0	2	1	27		
	More than 5 year but less than 10 years	0	1	2	37		

The findings of chi-square tests analyzing the link between many demographic variables and HCWs’ familiarity with several fit testing techniques at KSUMC are shown here. For profession and job experience, the study shows notable correlations, but not for hospital or department affiliation.

With φ^2^ = 17.948, *p* = 0.117, hospital affiliation shows no appreciable correlation with understanding of fit testing techniques. Comparably, department affiliation likewise shows no appreciable correlation (φ^2^= 30.851, *p* = 0.324). This implies that knowledge of fit testing techniques is quite constant throughout several departments and hospitals within KSUMC.

With respect to awareness of fit testing techniques, however, occupation shows a strong correlation (φ^2^ = 29.429, *p* = 0.021). Particularly with qualitative fit assessment, nurses show the best awareness among all the approaches. Doctors clearly reveal a difference between those who know about qualitative testing and those ignorant of such techniques. This suggests that the understanding of fit assessment differs among various medical professions; nurses are usually better educated.

Awareness of fit testing techniques (φ^2^ = 36.918, *p* = 0.002) is strongly correlated with length of work experience at KSUMC. HCWs with more than one year of experience exhibit greater understanding of qualitative fit assessment than those with less than one year of experience. Fascinatingly, knowledge of user seal checks and quantitative fit testing is somewhat lacking across all expertise levels. This implies that, especially for qualitative fit assessment, experience helps with awareness.

Although knowledge of quantitative testing and user seal checks is far lower, qualitative fit testing is the most often used approach across all categories. Not surprisingly, many of the respondents in all the groups claimed that they were not aware of any fit assessment techniques.

These results show that although knowledge of fit testing techniques is quite constant throughout hospitals and departments, it differs greatly depending on occupation and work experience. Particularly for quantitative testing and user seal checks, as well as for younger HCWs and non-nursing professionals, the results imply a need for more thorough education on fit testing approaches. Designing focused training courses to increase general awareness and knowledge of fit testing techniques among all HCWs at KSUMC might find great use from this information. See Table 5.

**Table 5:** Awareness of fit testing methods among healthcare staff at KSUMC by hospital, department, profession, and Experience.

		Are you aware of the different methods used for fit testing? (Select all that apply)					
		Qualitative fit test	Quantitative fit test	User seal check	No, I am not aware of any fit testing methods	Test Stat	P value
Hospital (Choose one answer)	DUH	14	3	2	4	17.948^a^	0.117
	GUC	2	2	0	0		
	KAUH	36	1	5	3		
	KKUH	89	8	9	17		
Department (Choose one answer)	Engineering	0	0	0	1	30.851^a^	0.324
	Laboratory	1	0	0	0		
	Medicine	10	2	1	4		
	Nursing	103	7	11	12		
	Others	17	5	3	2		
	Rehabilitation	0	0	0	1		
	Support service	1	0	0	0		
	Surgery	9	0	1	4		
What is your profession at KSUMC	Nurse	128	11	15	17	29.429^a^	0.021
	Others	7	3	1	1		
	Physician	6	0	0	4		
	Physiotherapist	0	0	0	1		
How long have you been working in a healthcare setting at KSUMC? (Choose one answer)	Less than 1 year	5	3	1	7	36.918^a^	0.002
	More than 1 year but less than 5 years	50	4	5	12		
	More than 10 years but less than 15 years	32	4	2	0		
	More than 15 years	25	0	3	2		
	More than 5 year but less than 10 years	29	3	5	3		

This information shows, in both respirator fit testing and PAPR use among healthcare personnel at KSUMC, correlation coefficients between demographic characteristics and confidence levels. Let me analyze every correlation. Department and confidence in PAPR usage show a modest but noteworthy positive association (*r* = 0.173, *p* = 0.05), suggesting that some departments could have somewhat greater confidence levels in PAPR use. Profession and confidence in PAPR use had a somewhat greater, significant positive connection (*r* = 0.213, *p* = 0.01), suggesting that confidence in PAPR usage differs greatly among several healthcare professions. Confidence in PAPR usage and confidence in respirator fit tests show a modestly negative connection (*r* = -0.287, *p* = 0.01). This intriguing result implies that confidence in one area increases, whereas confidence in the other often decreases. Although statistically nonsignificant, hospital affiliation reveals a modest negative connection with PAPR confidence (*r* = -0.104). Although this finding is similarly not statistically significant, length of work experience indicates a weakly favorable link with PAPR confidence (*r* = 0.110).

These results imply that the most important link is the negative association between confidence in fit testing and confidence in PAPR usage, even if profession and department somewhat affect confidence levels in PAPR use. This can point to a possible specialization effect whereby HCWs can grow more confident in one sort of respiratory protection at the expense of the other. Designing training courses aimed at developing balanced confidence across both forms of respiratory protection might find great use for this knowledge. Furthermore, departmental and professional variances in PAPR confidence point to the possible value of focused training programs. See Table 6.

**Table 6:** Correlations between confidence in fit testing knowledge and PAPR use across hospitals, departments, professions, and experiences at KSUMC.

	Hospital	Department	profession at KSUMC	How long have you been working in a healthcare setting at KSUMC?	How confident are you in your knowledge of the importance of regular fit testing for respirators?
How confident are you in your knowledge of the proper use of PAPR?	− 0.104	0.173^*^	0.212^**^	0.110	− 0.287**

** Correlation is significant at the 0.01 level (2-tailed). *. Correlation is significant at the 0.05 level (2-tailed).

Data on knowledge and attitudes concerning PAPRs among KSUMC HCWs indicate a combination of strengths and places for development.

Although not routinely, instruction on PAPR usage seems to be very common: 48%(N-94) of respondents receive regular training, and 34.2%(N-67) receive some training. This is shown in the confidence levels: 50% (N-98) of the respondents felt extremely confident in their knowledge of appropriate PAPR use, whereas 35.2% (N-69) felt just fairly confident. On the other hand, 14.8%(N-29) show poor confidence, which points to the need for more thorough training courses.

In general, PAPR component knowledge is excellent; 76.5%(N-150) of the respondents correctly noted that a PAPR system does not include a face mask. Analogously, 79.6%(N-156) accurately noted that the frequency of filter cartridge changes. However, only 55.6%(N-109) of the respondents could accurately determine the correct order for donning a PAPR, suggesting a need for more practical, hands-on instruction.

Regular inspections are clearly important, and 76.5%(N-150) of the respondents correctly said that PAPR equipment should be checked prior to every usage. Although there is some uncertainty regarding its capabilities, with just 12.3% (N-25) knowing it protects against both airborne particles and gases, the majority (64.2%(N-13)) agree that PAPR offers a better degree of respiratory protection than regular surgical masks do.

With 89.8% (N-176)ranking PAPR as extremely essential (4 or 5 on a 5-point scale), appropriate PAPR use helps reduce infectious disease spread. Although 22.4% (N-44) of the respondents considered PAPR not user friendly despite its great protection, most respondents (76%(N-149)) saw it as extremely protective, user friendly, and a decent alternative to N95 respirators.

In terms of actual practice, 24% (N-47) of the respondents said that they seldom or never used the correct PAPR at KSUMC, whereas 76%(N-149) felt that it was usually or largely used there. This disparity raises questions about the necessity of more uniform use of protocols and possibly greater supervision or reminders in clinical environments.

In particular, the influence of KSUMC on PAPR use appears to be rather unclear. Missing from the data the right response regarding PAPR being exclusively for individuals who failed the respirator fit test or whose fit-tested respirator size is unavailable indicates a gap in policy communication or knowledge.

Although PAPR is generally viewed favorably and there is a strong basis of knowledge, there are obviously some areas for development. These include clarifying institutional regulations, improving practical training on PAPR usage, attending to the usability issues of some personnel, and guaranteeing more uniform use of appropriate PAPR use procedures across all healthcare professionals at KSUMC. See Table 7.

**Table 7:** Assessment of knowledge and attitudes toward the proper use of the PAPR.

Assessment of knowledge and attitudes toward the proper use of PAPR	Frequency(*N*)	Percentage (%)
Which of the following responses is true about the proper use of PAPR? (Choose one answer)		
Use of PAPR is mandatory for all regardless of the availability of my proper size of fit tested respirator.	12	6.1%
Use of PAPR is necessary for all staff as a requirement for CBAHI and Canadian accreditation.	15	7.7%
Use of PAPR is only for those that failed the respirator fit test, or whose fit-tested respirator size is not available		
Use of PAPR is optional for all regardless of the availability of my proper size of fit tested respirator.	23	11.7%
Have you received training on the proper use of PAPR? (Choose one answer)		
No, and I am not interested in receiving training	7	3.6%
No, but I would like to receive training	28	14.3%
Yes, but not regularly	67	34.2%
Yes, regularly	94	48.0%
How confident are you in your knowledge of the proper use of PAPR? (Choose one answer)		
Not confident at all	6	3.1%
Not very confident	23	11.7%
Somewhat confident	69	35.2%
Very confident	98	50.0%
One of the following is NOT a component of a PAPR system: (Choose one answer)		
Blower	10	5.1%
Breathing tube	4	2.0%
Face mask	150	76.5%
Hood	10	5.1%
Waist belt	22	11.2%
How often should the filter cartridges in a PAPR system be changed? (Choose one answer)		
After each use	26	13.3%
At least once every 6 months or according to the manufacturer’s recommendations	156	79.6%
Once a month	7	3.6%
Once a week	7	3.6%
What are the correct steps for donning a PAPR? (Choose one answer) (A) Put on the face-piece (hood) and secure it tightly (B) Ensure the battery pack is fully charged, (C) Turn on the PAPR and ensure proper airflow, (D) Connect the filter cartridges to the face-piece, (E) Attach the breathing tube to the face-piece and battery pack		
ABCDE	32	16.3%
BCDEA	109	55.6%
CDEAB	39	19.9%
DEABC	16	8.2%
How often should PAPR equipment be inspected and maintained? (Choose one answer)		
Before each use	150	76.5%
It does not require regular inspection or maintenance	12	6.1%
Once a month	12	6.1%
Once a year	22	11.2%
What are the reasons for using a PAPR instead of a standard surgical mask? (Select all that apply)		
It provides a higher level of respiratory protection	131	64.2%
It protects against both airborne particles and gases	25	12.3%
It is more comfortable to wear for long periods	2	1.0%
It is cheaper than surgical masks	1	0.5%
None of the above	37	18.1%
How would you rate the importance of proper PAPR usage in preventing the transmission of infectious diseases?		
1	2	1.0%
2	1	0.5%
3	17	8.7%
4	29	14.8%
5	147	75.0%
Choose which option represents your view on the use of PAPR (Choose one answer)		
A. It is highly protective, user friendly, and a good alternative to the N95 respirator	149	76.0%
B. It is highly protective but not user friendly	44	22.4%
C. It not protective, but user friendly	2	1.0%
D. It is neither protective not user friendly.	1	0.5%
Do you believe that proper PAPR usage is consistently practiced by HCWs at KSUMC? (Choose one answer)		
No, never	5	2.6%
No, rarely	42	21.4%
Yes, always	91	46.4%
Yes, most of the time	58	29.6%

The findings of chi-square tests analyzing the link between certain demographic variables and HCWs’ reception of training on the correct use of PAPRs at KSUMC are shown here. Across all the demographic factors examined, the study revealed notable correlations.

PAPR training status (φ^2^ = 34.250, *p* < 0.001) is strongly correlated with hospital affiliation. While DUH has the lowest proportion, KKUH is the greatest among HCWs who have had consistent training. This suggests that variations may existing PAPR training accessibility or priority across many hospital sites within KSUMC.

With PAPR training status (φ^2^ = 55.045, *p* < 0.001), department affiliation also exhibits a noteworthy correlation. While certain areas, such as engineering and support services, have more variable replies, the nursing and medicine departments have the largest number of HCWs with regular training. This can point to variations in PAPR training demands or availability between many hospital departments.

With PAPR training status (φ^2^ = 62.645, *p* < 0.001), profession exhibited a very significant correlation. While other occupations exhibit more varied replies, nurses almost always indicate having had frequent PAPR training (92 out of 110). This implies that, among nursing professionals, PAPR training might be more regularly used or enforced than in other healthcare professions.

PAPR training status (φ^2^ = 23.127, *p* = 0.027) is strongly correlated with length of employment experience at KSUMC. HCWs with 1–5 years of experience have regular training; those with less than one year of experience have more diverse answers. This would suggest that throughout the first few years of work, PAPR training is more likely to follow the first on-boarding phase.

These findings generally show notable differences in PAPR training status depending on several demographic criteria at KSUMC. Strong correlations imply that whether an HCW has undergone PAPR training depends greatly on their department, profession, hospital affiliation, and work experience. These results can be helpful in identifying areas where PAPR training programs need to be standardized or upgraded throughout the healthcare system. See Table 8.

**Table 8:** Analysis of PAPR training among healthcare professionals at KSUMC by hospital, department, profession, and experience.

		Have you received training on the proper use of PAPR				Test statistics	P value
		No, and I am not interested in receiving training	No, but I would like to receive training	Yes, but not regularly	Yes, regularly		
Hospital	DUH	3	12	8	0	34.250^a^	0.000
	GUC	0	4	0	0		
	KAUH	22	20	3	0		
	KKUH	69	31	17	7		
Department	Engineering	0	0	1	0	55.045^a^	0.000
	Laboratory	0	1	0	0		
	Medicine	9	5	2	1		
	Nursing	76	41	15	2		
	Others	5	13	7	2		
	Rehabilitation	0	0	0	1		
	Support service	0	1	0	0		
	Surgery	4	6	3	1		
What is your profession at KSUMC	Nurse	92	57	18	5	62.645^a^	0.000
	Others	2	7	3	0		
	Physician	0	3	6	1		
	Physiotherapist	0	0	0	1		
How long have you been working in a healthcare setting at KSUMC?	Less than 1 year	6	6	3	1	23.127^a^	0.027
	More than 1 year but less than 5 years	34	20	14	3		
	More than 10 years but less than 15 years	22	15	2	0		
	More than 15 years	19	5	3	3		
	More than 5 year but less than 10 years	13	21	6	0		

The findings of chi-square tests analyzing the link between certain demographic variables and HCWs’ confidence levels in their understanding of the correct usage of PAPRs at KSUMC are shown here. Across all the demographic factors examined, the study revealed notable correlations.

PAPR confidence is significantly correlated with hospital affiliation (φ^2^ = 17.973, *p* = 0.035). While DUH indicates the lowest proportion, KKUH is the greatest among HCWs who are extremely confident or moderately confident in their understanding of PAPR use. This suggests that variations may exist in PAPR training quality or accessibility across many hospital sites within KSUMC.

PAPR confidence (ω^2^ = 26.044, *p* = 0.205) shows no appreciable correlation with department affiliation. This implies that, throughout the several hospital departments of KSUMC, trust in PAPR usage is very constant.

Profession indicates a strong correlation with PAPR confidence (φ² = 29.744, *p* = 0.003). While doctors clearly distinguish between those who are highly confident and those who are not very confident, nurses exhibit the highest degree of confidence. This finding shows that confidence in the PAPR differs depending on the medical field; nurses usually have greater confidence.

PAPR confidence (φ^2^ = 12.235, *p* = 0.427) is not strongly correlated with the length of employment at KSUMC. This implies that among HCWs at KSUMC, PAPR confidence is not strongly influenced by experience level.

These findings generally show notable differences in PAPR confidence depending on various demographic criteria within the KSUMC. The strong correlations between profession and hospital affiliation imply that these elements are essential in influencing the confidence of HCWs in PAPR application. These results may be helpful in identifying areas where focused training or support initiatives might help increase PAPR confidence among medical professionals. See Table 9.

**Table 9:** Confidence in proper PAPR use among healthcare staff at KSUMC by hospital, department, profession, and experience.

		How confident are you in your knowledge of the proper use of PAPR?				Test statistics	P value
		Very confident	Somewhat confident	Not very confident	Not confident at all		
Hospital	DUH	7	7	8	1	17.973^a^	0.035
	GUC	2	1	1	0		
	KAUH	24	19	1	1		
	KKUH	65	42	13	4		
Department	Engineering	0	0	1	0	26.044^a^	0.205
	Laboratory	0	1	0	0		
	Medicine	12	4	0	1		
	Nursing	72	44	14	4		
	Others	8	13	6	0		
	Rehabilitation	0	1	0	0		
	Support service	1	0	0	0		
	Surgery	5	6	2	1		
	Nurse	93	59	16	4	29.744^a^	0.003
	Others	3	5	4	0		
	Physician	2	4	2	2		
	Physiotherapist	0	1	0	0		
How long have you been working in a healthcare setting at KSUMC? (Choose one answer)	Less than 1 year	11	3	2	0	12.235^a^	0.427
	More than 1 year but less than 5 years	34	26	8	3		
	More than 10 years but less than 15 years	23	13	2	1		
	More than 15 years	16	11	2	1		
	More than 5 year but less than 10 years	14	16	9	1		

## Discussion

In line with Objective 1, our findings showed high compliance with fit testing (93.4% (N-183)), particularly among nurses. However, Objective 2 revealed substantial knowledge gaps: only 6.9%(N-14) of respondents were aware of quantitative fit testing methods, indicating limited understanding beyond qualitative approaches. Knowledge, attitudes, and behaviors regarding respirator fit testing and PAPR use among HCWs at KSUMC were investigated in this study. The results draw important attention to areas for possible development and offer insightful analysis of the present situation of respiratory protection policies in this hospital system.

### Testing respirators: compliance and awareness

With 93.4%(N-183) of the responders having a valid fit test certificate, our findings show a good degree of compliance with fit testing criteria among HCWs at KSUMC. This compliance rate stands out as being much greater than that reported in some earlier studies. For example, only 65% of the HCWs surveyed in 2019 had received fit testing during the prior year. The high level of compliance at KSUMC points to the efficient use of fit testing rules and procedures.

The study does, however, expose some knowledge vacuum concerning fit assessment techniques. Although 69.1%(N-141) of the respondents were aware of qualitative fit assessment, only 6.9%(N-14) were aware of quantitative techniques. Given that most of the medical city respondents (61.8%(N-110)) claimed to have undergone qualitative testing, this gap might be explained. Since quantitative testing might yield more exact findings, the lack of knowledge of quantitative techniques could affect the general efficacy of respiratory protection programs^4^.

### Variations in population

Fit testing status had notable correlations with certain demographic variables. Chi-square analysis revealed important correlations with hospital affiliation (φ^2^ = 18.835, *p* = 0.027) and department (φ^2^= 111.8<. In particular, nurses had the best degree of fit testing compliance. This finding is consistent with the results of previous studies, such as^8^, who reported that nurses were more compliant than other healthcare workers were.

The noted differences among hospitals and departments inside KSUMC reflect possible discrepancies in the fit testing program execution. Such differences might reveal the necessity of consistent policies all over the healthcare system and cause gaps in respiratory protection.

### Attitudes and knowledge

Most of the respondents show great faith in their understanding of the need for frequent fit testing. Maintaining high compliance rates and guaranteeing the efficacy of respiratory protection programs depend on this good attitude^3^.

Nevertheless, several knowledge holes were found. For example, only 48.4%(N-93) of the respondents appropriately noted KSUMC’s single-use respirator policy. This misinterpretation might result in incorrect respirator reuse, therefore reducing their efficacy and increasing the risk of respiratory infection.

### When a powered air-purifying respirator (PAPR) is used, confidence and training are vital

Among the responders, 82.2% (N-168) had some kind of PAPR training; 48%(N-168) of them had regular instructions. This somewhat high training rate is positive, as efficient usage of PAPRs depends on appropriate training^7^. Nevertheless, the fact that 17.9%( N-35) of the respondents said that they had not had any instruction suggests that more thorough training programs are needed.

With 85.2%(N-176) of the respondents feeling very or very confident, PAPR usage confidence levels were typically high. Appropriate and consistent PAPR usage in clinical environments depends on this confidence. Nevertheless, 14.8%(of N-20) of the respondents who showed poor confidence constituted a sizable fraction that would gain from more help or training.

### Knowing PAPR Components and Use

Although most people knew about PAPR components—76.5% of them correctly noted that a face mask is not a component of a PAPR system—only 55.6% could properly identify the appropriate sequence for donning a PAPR. This disparity between theoretical understanding and actual experience emphasizes the necessity of additional practical, hands-on training in PAPR use.

Most of the responders (79.6%(N-156)) accurately indicated the frequency of filter cartridge changes, indicating strong knowledge of maintenance needs. However, obvious uncertainty about KSUMC’s PAPR strategy indicates a need for better institutional policy communication.

### Opinions About PAPR Use

With 89.8%(N-176) of respondents evaluating PAPR use as extremely essential, the value of appropriate PAPR use in stopping infectious disease transmission is widely known. Encouragement to adhere to appropriate PAPR use guidelines depends on this positive attitude^1^.

Most responders (76%(N-149)) thought the PAPR was an excellent substitute for N95 respirators, was very protective, and was user friendly. Nevertheless, 22.4% (N-44)thought it was user friendly even with great security. This view of poor usability might result in lower compliance with PAPR usage in cases when it is advised or mandated.

### Demographic Variations in PAPR Training: Confidence

PAPR training status and demographic variables were significantly correlated. With hospital affiliation (φ^2^ = 34.250, *p* = 0.001), department (φ^2^= 55.045, *p* = 0.027), profession (φ^2^ = 62.645, *p* = 0.027), and work experience (φ^2^ = 23.127, *p* = 0.027), chi-square analysis indicated important correlations. Nurses had the greatest rates of consistent PAPR training, much like in fit testing. These differences point to possible discrepancies in PAPR training initiatives spread throughout several parts of the healthcare system.

Given that nurses usually display greater confidence levels, confidence in PAPR usage also differed greatly between hospitals (φ^2^ = 17.973, *p* = 0.035) and professionals (φ^2^= 29.764, *p* = 0.003). However, job experience and PAPR confidence (φ^2^ = 12.235, *p* = 0.427) showed no appreciable correlation. These differences emphasize the necessity of more uniform training methods and can result in different PAPR use practices throughout the healthcare system.

### Fit testing compared with PAPR knowledge

Confidence in PAPR usage and confidence in respirator fit testing showed an interesting modest negative connection (*r* = − 0.287, *p* = 0.01). This negative connection points to a possible specialist effect whereby HCWs may grow more confident in one kind of respiratory protection at the expense of the other. This result highlights the need for thorough training courses that include both types of respiratory protection equally.

### Implications

The results of this study imply various important consequences for the practice of healthcare, especially with respect to the use of respiratory protection. Although general fit testing compliance is excellent, there are still notable knowledge gaps, particularly with respect to quantitative fit testing techniques and institutional respirator usage. This emphasizes how important focused instructional interventions are in closing these disparities. The study also identified differences in fit testing and powered air-purifying respirator (PAPR) training among many hospitals, departments, and professional groups, underscoring the need to standardize training programs and policies throughout the healthcare system.

The need to include additional hands-on, practical training sessions in the curriculum is shown by a clear difference between theoretical knowledge and practical abilities in PAPR usage points. Moreover, the inverse link found between PAPR use and confidence in fit testing highlights the importance of balanced training courses that equally prioritize both types of respiratory protection. Finally, the view of PAPRs as not user friendly by a considerable minority of healthcare professionals points to the necessity of resolving usability problems and providing extra help to guarantee the efficient use of these devices.

### Limitations

One should admit the numerous limitations of this study. First, it depends on self-reported data, which by nature suffer social desirability bias and recall bias, therefore compromising the validity of the results. Although the sample size was significant (*N* = 204), the generalizability of the results may be limited if the sample size does not reflect all healthcare professionals (HCWs) at KSUMC. Furthermore, the study lacked observational techniques to evaluate real-world behavior, thereby offering less objective information on adherence to respiratory protection guidelines. Finally, the cross-sectional design of the study limits the capacity to create causal links between demographic elements and the knowledge, attitudes, or behaviors of HCWs with respect to respiratory protection.

### Recommendations

Based on our findings, we recommend the following:

1. Develop standardized, cross-departmental respiratory protection training programs that equally emphasize both respirator fit testing and PAPR use, including hands-on practical sessions to improve procedural knowledge.
2. Improve institutional policy communication, especially regarding fit testing frequency, respirator reuse rules, and indications for PAPR use, to address the observed inconsistencies in understanding.
3. Prioritize quantitative fit testing education, as knowledge of this method was notably low, despite its critical role in ensuring optimal respirator effectiveness.
4. Address confidence gaps among non-nursing professionals, through targeted training and mentoring programs that leverage the strengths and experiences of high-confidence HCWs.
5. Conduct periodic evaluations of respiratory protection knowledge and practices to ensure that compliance remains high and emerging gaps are promptly addressed.

## Conclusions

This study provides a comprehensive assessment of HCWs’ knowledge, attitudes, and practices regarding respirator fit testing and PAPR use at KSUMC. While compliance with fit testing protocols was high, key knowledge and training gaps were identified—especially regarding quantitative testing methods and correct PAPR procedures. Confidence levels and training exposure varied by profession and department, underscoring the need for standardized, inclusive training programs. The results reinforce the importance of clear policy communication and hands-on instruction to ensure preparedness and occupational safety among healthcare professionals. This study provides a comprehensive evaluation of HCWs’ knowledge, attitudes, and practices related to respirator fit testing and PAPR use at KSUMC. While the majority of HCWs demonstrated high compliance with fit testing protocols (93.4%), significant knowledge gaps were observed—particularly in awareness of quantitative fit testing methods, with only 6.9% identifying them correctly. PAPR usage was widespread (85.2%), but confidence and proper donning procedures varied considerably among participants. Moreover, disparities in training access and confidence levels were evident across professions and departments, with nurses reporting higher exposure to training and greater confidence than other HCWs.

The data also revealed a modest but significant inverse relationship between confidence in fit testing and confidence in PAPR use, suggesting a need to balance training efforts across both areas of respiratory protection.

## Data Availability

All the data generated or analyzed during this study are included in this published article.
